# Author Correction: Simulating the ghost: quantum dynamics of the solvated electron

**DOI:** 10.1038/s41467-021-21706-2

**Published:** 2021-02-22

**Authors:** Jinggang Lan, Venkat Kapil, Piero Gasparotto, Michele Ceriotti, Marcella Iannuzzi, Vladimir V. Rybkin

**Affiliations:** 1 Department of Chemistry, University of Zurich, Zürich, Switzerland; 2 Laboratory of Computational Science and Modelling, Institute of Materials, Ecole Polytechnique Fédérale de Lausanne, Lausanne, Switzerland; 3 Empa, Swiss Federal Laboratories for Materials Science and Technology, Dübendorf, Switzerland

**Keywords:** Chemistry, Electron transfer, Computational chemistry, Molecular dynamics

Correction to: *Nature Communications* 10.1038/s41467-021-20914-0, published online 3 February 2021.

The original version of this Article contained an error in the spelling of the author Jinggang Lan, which was incorrectly given as Jingang Lan.

This has now been corrected in both the PDF and HTML versions of the Article.

