# Supplementary material for: Simulating the ghost: quantum dynamics of the solvated electron
Source: Nat Commun. 2021 Feb 3;12:766. doi: 10.1038/s41467-021-20914-0 (PMC7859219; doi:10.1038/s41467-021-20914-0)
Supplement: Supplementary file 1 — Supplementary Information [file 41467_2021_20914_MOESM1_ESM.pdf]

Supplementary Materials for
Simulating the Ghost: Quantum Dynamics of the Solvated Electron

Jingang Lan,[1,*] Venkat Kapil,[2] Piero Gasparotto,[3] Michele Ceriotti,[2] Marcella Iannuzzi,[1] and Vladimir V. Rybkin[1,†]

[1]*Department of Chemistry, University of Zürich, Zürich, Switzerland*
[2]*Laboratory of Computational Science and Modelling, Institute of Materials, Ecole Polytechnique Fédérale de Lausanne, Lausanne 1015, Switzerland*
[3]*Empa, Swiss Federal Laboratories for Materials Science & Technology, 8600 Dübendorf, Switzerland*

(Dated: December 17, 2020)

* jinggang.lan@chem.uzh.ch
† vladimir.rybkin@chem.uzh.ch

I. SUPPLEMENTARY METHODS

A. Computational methods

The MP2 simulations have been performed using a cubic cell with a length of 11.295 Å containing 47 water molecules, which is sufficient to reproduce essential observables from experiments.[1] Spin-restricted and spin-unretricted MP2 calculations [2, 3] with triple-zeta quality correlation-consistent basis sets [4] have been carried out using CP2K,[5, 6] which have further been used as training data for the BPNN potential [7]. The training set is based on 14185 MP2 energies and forces for bulk water and 2798 UMP2 energies and forces for solvated electron. More details of electronic structure calculations and BPNN are given below.

All MD simulations are performed by using I-PI code[8] interfaced with the LAMMPS [9] code that implements the BPNN potential. To achieve efficient canonical sampling while minimally perturbing the dynamics, the classical molecular dynamics have been performed using a stochastic velocity-rescaling (SVR) thermostat[10] with a timestep of 0.5 fs and constant temperature sampling enforced with a time constant of 1000 fs.

Quantum molecular dynamics has been carried out using thermostated ring-polymer molecular dynamics (TRPMD) method with a timestep of 0.25 fs and PILE-G thermostat with time constant of 1000 fs .[11]. We used 32 beads for the PIMD simulations, which is sufficient to converge quantum kinetics at room temperature. TRPMD is designed for computation of dynamical properties. In particular, it has been demonstrated to predict accurate vibrational dynamics of liquid water.[12, 13] The TRPMD gives satisfying spectra that line up with the quasi-centroid molecular dynamics spectra which is the best available method for reproducing fundamental transitions in the spectrum. [12, 13] All the e^-(aq) simulations started from different water structures after 15 ps equilibrium and last 10 ps. Based on trajectories as obtained from BPNN, we visualize the spin-density every 10 fs by using hybrid density functional theory with PBE(α) hole with 40%-HFX using the auxiliary density matrix methods (ADMM)[14] as implemented in CP2K, which predicts a comparable spin density compared to MP2 and also yields a Kohn-Sham band gap of 8.8 eV for liquid water. [15, 16]

B. MP2 calculations

The MP2 correlation energies and forces have been computed within the resolution-of-identity approximation in the Gaussian and plane waves framework [2, 3]. The truncated Coulomb operator [17] has been applied for the exchange calculations with the cutoff radius approximately equal to half the length of the smallest edge of the simulation cell, 5.62 Å, together with the Schwarz integral screening with the threshold of 10^{-10} a. u. SCF convergence criterion was set to $5 \cdot 10^{-7}$ a.u. The plane waves cutoff for the Hartree-Fock part of the calculations was 500 Ry, whereas the cutoff for the correlation energy calculations was 300 Ry, respectively. Atomic core electrons are described through Goedecker-Teter-Hutter (GTH) pseudopotentials [18, 19] optimized for Hartree-Fock theory. For this system size, each the energy and force calculation on 256 hybrid CPU/GPU compute nodes took 12-16 minutes of wall-time.

C. Machine learning

The construction of training set is of great importance to make an accurate potential. The dataset obtained from AIMD are recycled from Ref [20] using MP2 for water and Ref [1] using UMP2 for solavted electron. The reference data obtained using the classical molecular dynamics approach may be highly correlated and insufficient, failure of MD based on first machine learning potential may occur. Besides, the quantum fluctuations region haven't been visited using classical molecular dynamics sampling method, the machine learning potential may extrapolate those regions without full guarantee. Therefore, retraining the potential is required, especially for those configurations far from the current datasets. To this end, a large set of configurations are generated using machine learning potential. The machine learning potential has been gradually improved by generating more points based on machine learning potential, selecting representative snapshots and recalculating at the MP2 level of theory then adding to the datasets. The cross validation has been performed at each iteration. And 100 additional structures for water and 600 additional structures for solvated electron are selected based on their atomic fingerprints.[21]

In our training set, there are 14085 configurations as obtained from classical molecular dynamics [20] and 100 additional representative configurations from quantum molecular dynamics for the bulk water. For the solvated electron, a total of 2798 configurations of solavted electron in water have been used, where 2198 structures are recycled from classical molecular dynamics Ref [1] and 600 additional structures. A detailed comparison between the machine learning potential and (U)MP2 energies are plotted in the Supplementary Figure 1. A root-mean-square

error(RMSE) of 2.19 meV/H_2O in energies and 90.04 meV/Å in forces are obtained for the bulk water; while a RMSE of 5.72 meV/H_2O in energies and 212.29 meV/Å in forces for the solvated electron.

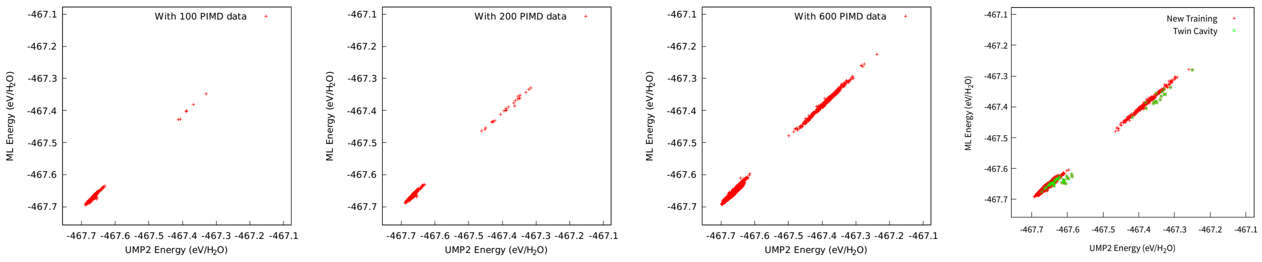

Supplementary figure 1: A comparison between the Machine learning energies computed using lammps, and the energies computed using UMP2 for solvated electron. From left to right are energy validation including 100, 200, 600 PIMD data and additional 100 twin-cavity data. The additional twin-cavity structure are highlighted in green crosses. The lower energy region are classical dataset from MD and high energy region are quantum dataset from PIMD

The selection of dataset is based on the atomic fingerprints method [21]. The first machine learning potential has been trained based on the previous data. Based on the first machine learning potential, we generate many molecular dynamics trajectories (About 60,000 snapshots) from classical and quantum dynamics. We then choose 100 representative snapshots and recalculate with MP2. The machine learning potential has been gradually improved by generating more points based on machine learning potential, selecting representative snapshots and recalculating at the MP2 level of theory then adding to the datasets. The cross validation has been performed at each iteration.

In the last plot of Supplementary Figure 1, we cross-validated the model on 100 additional twin-cavity points (marked in green crosses). The quality of our machine learning potential turned out to be reliable although energies of a few twin-cavity structures from the centroid are slightly underestimated by our MLP. However, RPMD, in principle, will not be able to "see" those points as the beads correspond to the data from PIMD datasets.

Furthermore, we trained a new machine learning potential which included the additional cross-validation twin-cavity dataset. We can still see the transient diffusion mechanism as proposed with the same frequency. No additional analysis on these simulations has been performed as they do not convey new information but serve the validation purpose only.

D. Hybrid-functional calculations

Based on ML trajectories, we visualize the spin-density every 10 fs using hybrid density functional theory with PBE(α) hole with 40%-HFX (see Eq.1), which has been proved to predict stratifying description for solvated electron[15, 16]. Molecular orbitals of the valence electrons are expanded in TZV2P basis sets[22], while atomic core electrons are described through Goedecker-Teter-Hutter (GTH) pseudopotentials [18, 19]. In Supplementary Figure 2, we plotted the spin density obtained from UMP2 and PBE(α) hole with 40%-HFX methods. Both methods predict very similar spin cavity for the solvated electron.

$$E_{\mathrm{xc}}^{\mathrm{PBEh}(\alpha)} = \frac{2}{5}E_{\mathrm{x}}^{\mathrm{HF}} + \frac{3}{5}E_{\mathrm{x}}^{\mathrm{PBEh}} + E_{\mathrm{c}}^{\mathrm{PBEh}} \quad (1)$$

E. Volumetric data analysis and processing

1. Calculation of distribution centre and gyration radius

Since the position operator $\mathbf{r}$ is not defined under periodic boundary conditions the gyration radius is not defined either. However, the simulation periodic cell is large enough to allow the treatment of periodic spin as non-periodic. For this purpose, the .cube files have been centered so that the spin density decays to zero on the boundaries of the cell.

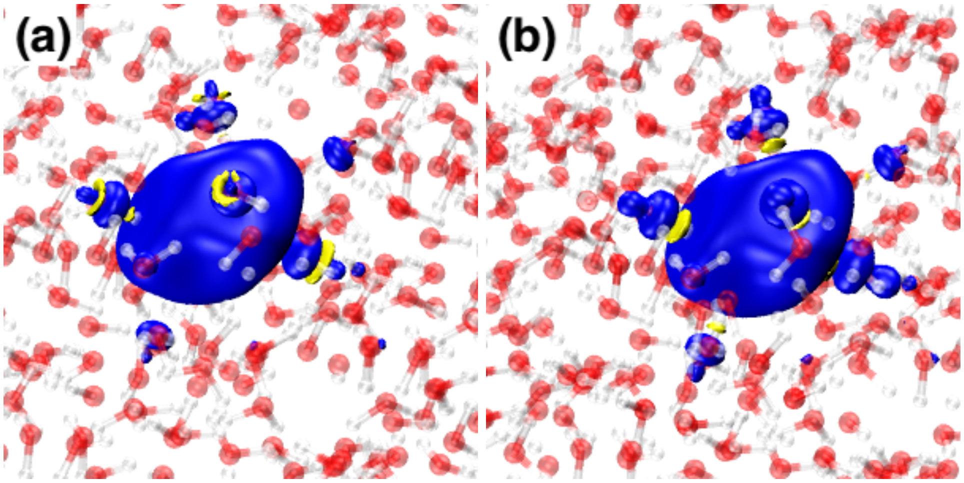

Supplementary figure 2: The spin density as obtained from (a) UMP2 and (b) PBE(α) hole with 40%-HFX methods. Color code: white(white), oxygen(red), Isosurface of 0.001 for spin density (blue), Isosurface of -0.002 for negative spin density (yellow)

The centre of spin density distribution $\rho^s(\mathbf{r})$ is given as follows:

$$\mathbf{r}_c = \int \rho^s(\mathbf{r})\mathbf{r}d\mathbf{r} \tag{2}$$

The second moment tensor reads:

$$\mathbf{S} = \int (\mathbf{r}-\mathbf{r}_c)(\mathbf{r}-\mathbf{r}_c)\rho^s(\mathbf{r})d\mathbf{r} \tag{3}$$

Then the gyration radius is calculated as:

$$r_g = \sqrt{\lambda_1^2+\lambda_2^2+\lambda_3^2}, \tag{4}$$

where λ_1, λ_2, λ_3 are eigenvalues of $\mathbf{S}$.

With the spin density on the real-space grid:

$$\mathbf{r}_c = \sum_{i=N}^{N} \rho^s(\mathbf{r})\mathbf{r}, \tag{5}$$

the elements of $\mathbf{S}$ are calculated as follows:

$$S_{xx} = \sum_{i=1}^{N}[(y_i-y_c)^2+(z_i-z_c)^2]\rho^s(\mathbf{r}_i), \tag{6}$$

$$S_{yy} = \sum_{i=1}^{N}[(x_i-x_c)^2+(x_i-x_c)^2]\rho^s(\mathbf{r}_i), \tag{7}$$

$$S_{zz} = \sum_{i=1}^{N}[(x_i-x_c)^2+(y_i-y_c)^2]\rho^s(\mathbf{r}_i), \tag{8}$$

$$S_{xy} = -\sum_{i=1}^{N}[(x_i-x_c)^2+(y_i-y_c)^2]\rho^s(\mathbf{r}_i), \tag{9}$$

$$S_{xz} = -\sum_{i=1}^{N}[(x_i-x_c)^2+(z_i-z_c)^2]\rho^s(\mathbf{r}_i), \tag{10}$$

$$S_{yz} = -\sum_{i=1}^{N}[(y_i-y_c)^2+(z_i-z_c)^2]\rho^s(\mathbf{r}_i), \tag{11}$$

where the summation runs over the N grid points.

2. Radial distribution functions, gyration radii and diffusion coefficients for classical and quantum MD

The centre of spin density distribution (the first moment) $\mathbf{r}_c$ was used as a position of the solvated electron for calculating radial distribution functions (RDFs) and diffusion coefficients.

In principle, the properties should be computed from the spin density distributions of the beads. To save computational resources, for only 2 quantum simulations we computed spin density distributions for 8 beads. For all quantum simulations spin density distributions were obtained for centroids. Below, we distinguish the values computed from the beads and from the centroids.

Solvated electron RDF in the case of quantum simulations are computed from 2 simulations (overall 20 ps), for which the spin densities were computed for 8 beads. For classical MD, RDFs are computed from spin density distributions of centroids.

Diffusion coefficients for quantum simulations are calculated from spin density distributions of centroids. This quantity computed from the beads are identical to those computed from the centroids within error margins.

We have found that the gyration radii from beads are 0.05 and 0.1 Å larger than those from the corresponding centroids for the two quantum simulations, where these data are available. Therefore, we added 0.1 Å to the rest of the values computed from centroids and 0.05 Å uncertainty to the error of the gyration radius in the quantum case.

Diffusion coefficients calculated from classical and quantum dynamics are within their statistical uncertainty of each other (0.36 ± 0.04 Å^2/ps and 0.40 ± 0.03 Å^2/ps, respectively), With the finite-cell correction estimated to be 0.07 the values become 0.36 ± 0.04 and 0.40 ± 0.03, and in good agreement with the experimental values of 0.490 ± 0.003 [23, 24] and 0.475 ± 0.048 Å^2/ps [25].

II. SUPPLEMENTARY DISCUSSION

A. Twin-cavity

The solvated electron can shuttle between two voids via the twin-cavity intermediate. The extent, to which the excess electron occupies both cavities in the intermediate, depends on the amount of exact exchange in the hybrid functional. As shown in Supplementary Figure 3, MP2 spin density distribution can be more compact than this from PBEh(40). In Supplementary Figure 3(a,b), for the pre-solvated cavity has and twin-cavity PBEh(40) predicts more diffuse spin density distributions. Importantly, for the latter, we observe the distribution with two peaks (both halves of the twin-cavity filled) at the PBEh(40) level and with one peak at the MP2 level (one half of the twin-cavity filled). After 20 fs the electron moves completely to the other half-cavity for both MP2 and PBEh(40) calculations as shown in the Supplementary Figure 3(c). It turns out that PBEh(40) functional, being a good proxy to MP2 for single cavity structures, is suboptimal for the twin-cavity structures.

Notwithstanding, this discussion does not affect the fact that we do observe transient diffusion and double cavities (even if both halves are not filled simultaneously).

The calculated anisotropy of double cavity is not that much at MP2 level is not much different from the single one. For instance, the anisotropies for the structures in Supplementary Figure 3(a,b,c) are 0.1234 and 0.0736 and 0.0438 from MP2, while 0.6000, 0.8482 and 0.0756 from PBEh(40) theory.

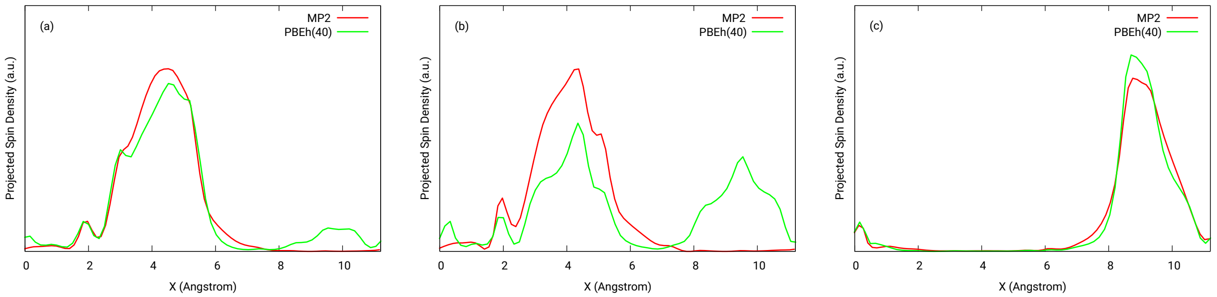

Supplementary figure 3: Projected spin density along X direction. The spin density are calculated using MP2(red) and PBEh(40)(green): (a) - pre-solvated cavity; (b) - twin-cavity; (c) - single cavity.

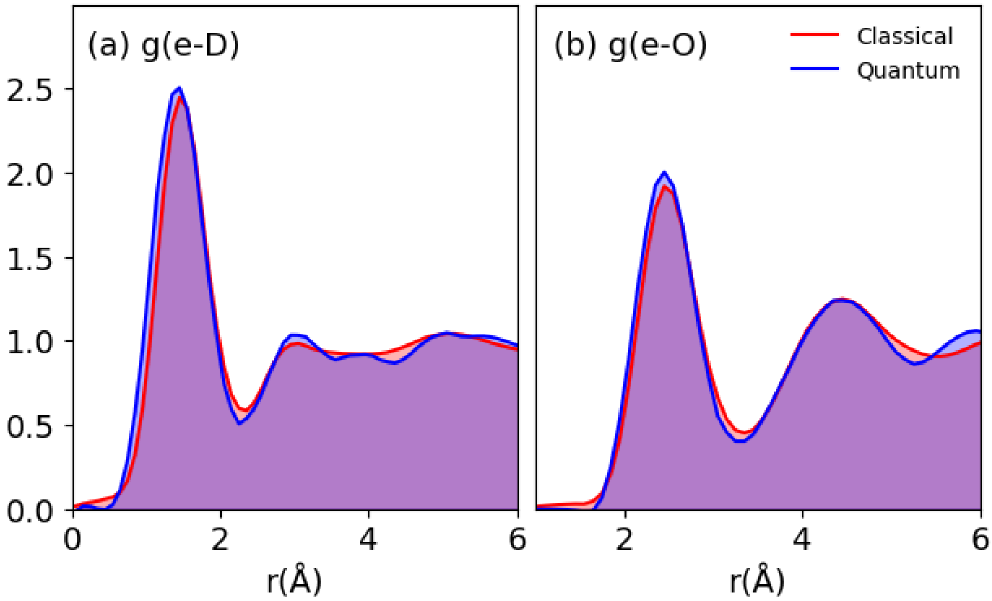

Supplementary figure 4: Radial distribution functions of deuterium (a) and oxygen (b) atoms from heavy water with respect to the center of the solvated electron (spin density distribution) from classical (red) and quantum (blue) molecular dynamics of water.

B. Radial distribution functions

In Supplementary Figure 4, we plotted the radial distribution functions of deuterium and oxygen atoms from heavy water with respect to the gyration center of the solvated electron. The RDF as obtained from the classical and quantum dynamics are almost identical. The contribution of the NQEs in heavy water is rather modest compared to water.

C. H-bond species populations

In this work, we use a machine learning approach to obtain a statistical definition of the atomic-scale motif representing the hydrogen-bond (HB) in water, based on a fully data-driven analysis. In the specific, we used the Probabilistic Analysis of Molecular Motifs (PAMM) algorithm [26, 27], which casts the probability density function (PDF) of structural data sampled from simulations, or experiments, into a Gaussian mixture model (GMM). PAMM has already been applied to the identification of HBs in water, ammonia [26], and proteins [28] and have demonstrated capable of capturing the structural differences between HBs characteristic of different water models, e.g. quantum and classical water [29].

The first step in the analysis consists of the definition of the groups of atoms that are typically involved in an HB, which in the case of water is fairly obvious: two distinct oxygens should be considered as donor D and acceptor A respectively, while a hydrogen atom H is needed to complete the HB triplet. As described in details in ref. 26, we define as input features for PAMM a set of $3D$ vectors derived from the combinations of the three interatomic distances (r_{DH}, r_{AH}, r_{AD}) from all the possible O–H–O triplets: the proton-transfer coordinate $\nu = r_{DH} - r_{AH}$, the symmetric stretch coordinate $\mu = r_{DH} + r_{AH}$ and the acceptor-donor distance $r = r_{AD}$. We then compute the (ν, μ, r) vector corresponding to each triplet in the simulation box of 30 independent snapshots sampled from 10 ps-long trajectories. Finally, we perform a kernel density estimation (KDE) on a sparse grid of 6000 points obtained by subsampling the input data with farthest point sampling (FPS) [27, 30, 31] and use a density-based clustering to identify the local maxima and eventually partition the initial triplets into distinct clusters. These clusters are used to build a GMM, provding us with a probabilistic framework able to associate regions of the 3D space to different recurring patterns recurring in the HB network. More specifically, once identified the cluster corresponding to HB patterns, we use the posterior probability of the corresponding Gaussian to obtain a natural, fuzzy definition of the HB, i.e. a function of the atomic coordinates that takes a value between zero and one and represents the degree of confidence by which a structure can be labeled as HB. All the analyses were done using the PAMM implementation available at the link

https://github.com/cosmo-epfl/pamm.

We exploit the ability of PAMM to identify the O–H–O triplets matching the HB pattern for counting the number of HBs involving each molecule as the sum over all the HB PAMM classifiers of the O–H–O triplets where the molecule's atoms take part to. This allows to represent the HB network as a directed graph where the defective state of each water molecule in the network is defined as in ref. 29. More specifically, we use two different types of HB counting functions: s_D that quantifies the total number of HBs donated by O atom and s_A the number as HBs accepted. For instance, the region with $1.5 \leq s_D < 2.5$ and $1.5 \leq s_A < 2.5$ is assigned to the 2_D2_A state. Based on these functions, we can define the hydrogen bond species population and produce the bar plots shown in Fig. 5.

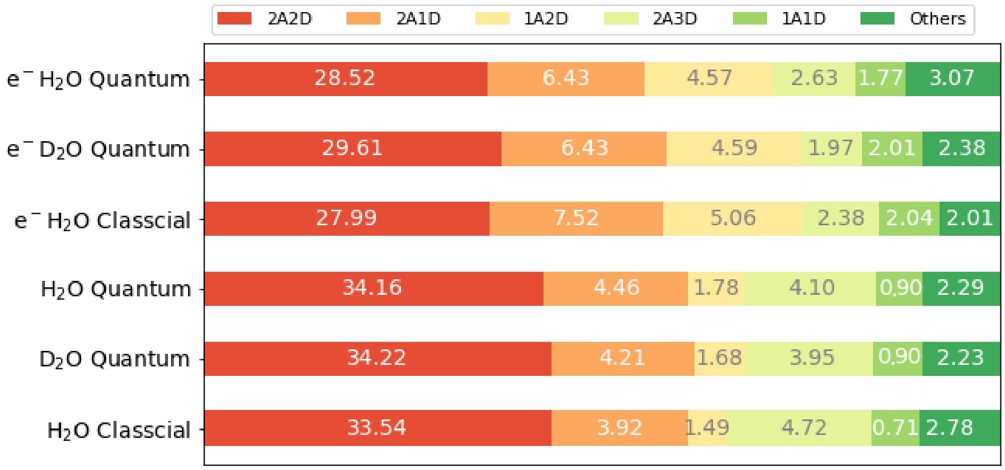

Supplementary figure 5: H-bond coordination population (number of water) for solvated electron (top) and water (bottom) at T = 300K as obtained from simulations. The presence of the electron perturbs greatly the H-bond network, decreasing the population of fully-tetrahedral (2_D2_A) molecules. For instance, the transition $2_D2_A \rightarrow 1_D2_A$ contributes to about $150\,\mathrm{cm}^{-1}$ downshift in the RR spectra, which explains the red shift in the O-H stretching region as discussed in the main text.

D. Vibrational density of states

The Vibrational density of states from an classical MD or TRPMD have been calculated by the Fourier transform of their velocity autocorrelation

$$C_{vv}(\omega) = \int \langle v(\tau)v(t+\tau)\rangle_\tau \, e^{-i\omega t} dt. \tag{12}$$

The power spectra has been averaged over 30 trajectories and first 2.5 ps have been skipped. Since the position (or velocity) is a linear operator, its average over beads is same as the centroid. Therefore, we used centroid the obtain the spectra.

For other properties, e.g. the band gap and gyration radius of the cavity, the analysis only on the centroid does not strictly apply. Instead, the analysis of all beads for all trajectories is in principle necessary, but practical not worthy.

E. Electronic spectra

We have computed electronic spectra of single- and double-cavities using time-dependent density functional perturbation theory (TDDFPT), often referred to as time-dependent DFT (TDDFT). We used with PBE(α) hole with 50%-HFX, producing spin density distributions similar to those of MP2 for both cavity types. We used 106 and 140 snapshots for single- and double-cavities, respectively.

Considering the first excited state for a single cavity, the largest excitation amplitude corresponding to the p-type orbital (see Supplementary Figure 7). However, the second and third excited states, corresponding to the other two p-orbitals found not for all structures. Instead, spurious delocalized states arise as described by Lange and Herbert

[32] due to finite cell size and as an intrinsic problem with TDDFT, even with hybrid functionals. Thus, we have computed the spectrum of the single cavity, having combined all excitations identified as s-p. This produces a wide band with a maximum around 2.2 eV (see Supplementary Figure 6). This spectrum is considerably blue-shifted and also wider than the experimental one ([33]). This, however, qualitatively agrees with the previously accumulated knowledge about solvated electon's electronic spectrum [34], proving that the system size is adequate and the methods are reasonable.

The spectrum of the double-cavity is also a broad continuous distribution with the maximum red-shifted as compared to the single cavity (see Supplementary Figure 6), after removing several spurious excitations. All other excitations correspond to the charge transfer between the two cavities as shown in Supplementary Figure 7. Keeping in mind the ration between single- and double-cavities as 9:1, we can conclude that the latter have little effect on the overall electronic absroption (see Supplementary Figure 6). We note that 10% content of double cavities is an upper bound: only three simulations out of 40 exhibit this trait and in all of them double-cavities alternate with the single ones.

Nevertheless, the twin-cavities must demonstrate bleaching of the ground state in the hole-burning experiments due to the asymmetry of the double void [35]. This signal, however, must be weak due to low concentrations of the double-cavity and should be observed in the first picoseconds after creation of the solvated electron as the double-cavity is a metastable, although persistent structure. Moreover, it appears promising to apply pulses of lower frequency corresponding to the maximum of the twin-cavity spectrum (below 1 eV).

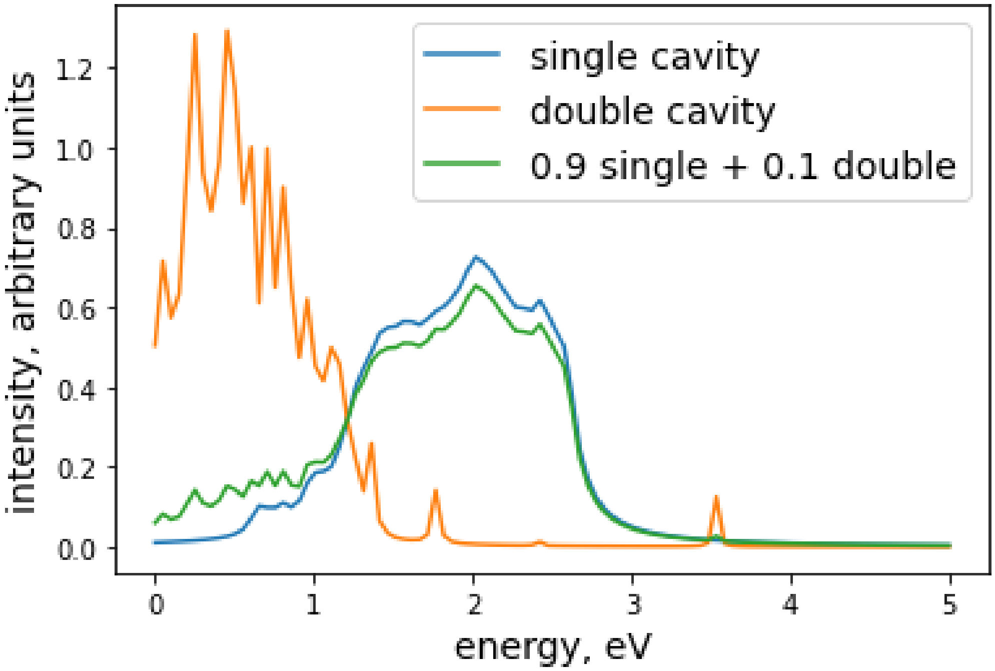

Supplementary figure 6: Electronic spectrum of the hydrated electron: single cavity, double cavity and mixed according to their concentration. Spurious charge-transfer states have been removed. Only excited states corresponding to s-p transitions are included for signle cavity. Only the first excited states are included for the double-cavity. Spectral line shapes were approximated with the Lorentz functions.

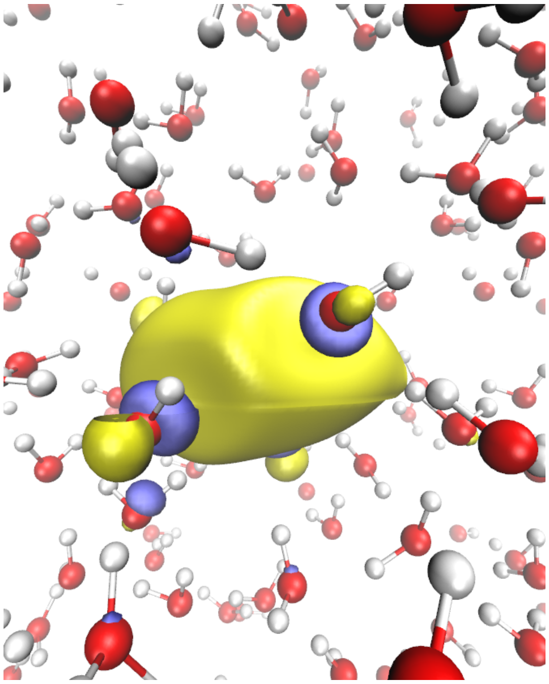

(a) SOMO of the single cavity (s-like type).

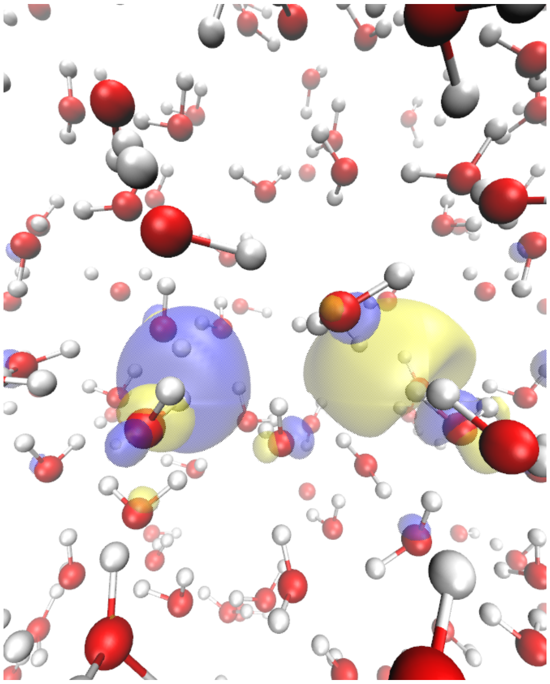

(b) LUMO of the single cavity (p-like type).

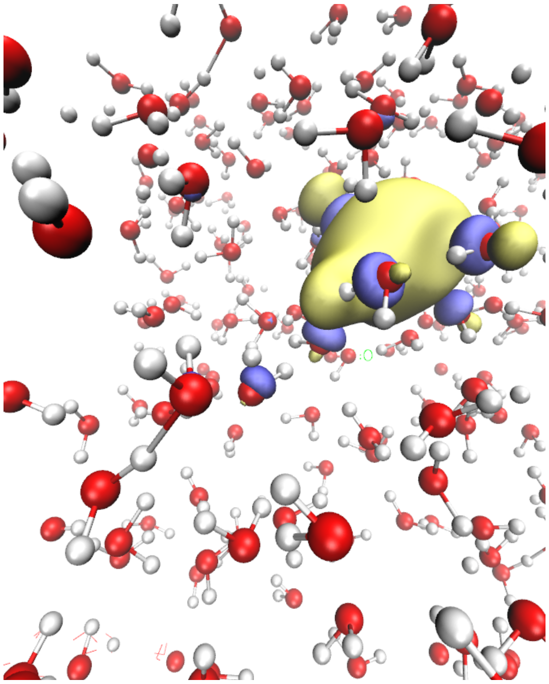

(c) SOMO of the double cavity (s-like type).

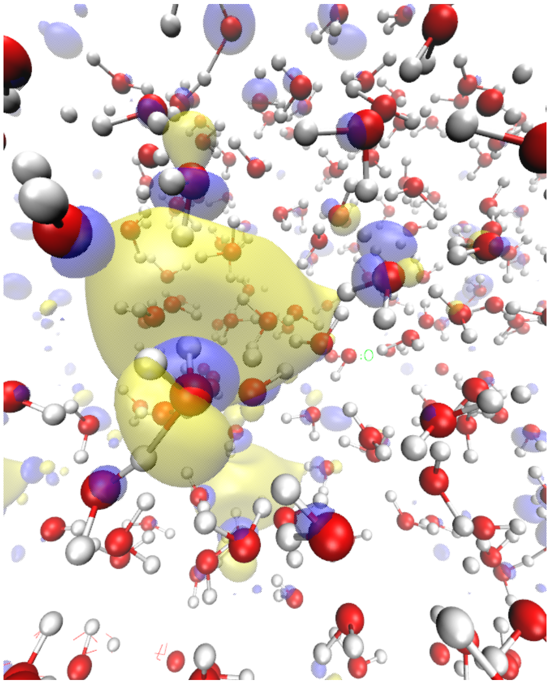

(d) LUMO of the double cavity (s-like type in the second subcavity).

Supplementary figure 7: Spin-orbitals responsible for the first electronic transition in single- and double-cavity structures. Blue: positive values, yellow: negative. The isovalue is ±0.03 a.u.

III. SUPPLEMENTARY REFERENCES

[1] J. Wilhelm, J. VandeVondele, and V. V. Rybkin, Angewandte Chemie International Edition **58**, 3890 (2019).
[2] M. Del Ben, J. Hutter, and J. VandeVondele, The Journal of Chemical Physics **143**, 102803 (2015).
[3] V. V. Rybkin and J. VandeVondele, Journal of Chemical Theory and Computation **12**, 2214 (2016).
[4] M. Del Ben, J. Hutter, and J. VandeVondele, Journal of Chemical Theory and Computation **9**, 2654 (2013).
[5] J. Hutter, M. Iannuzzi, F. Schiffmann, and J. VandeVondele, Wiley Interdisciplinary Reviews: Computational Molecular Science **4**, 15 (2014).
[6] T. D. Kühne, M. Iannuzzi, M. Del Ben, V. V. Rybkin, P. Seewald, F. Stein, T. Laino, R. Z. Khaliullin, O. Schütt, F. Schiffmann, D. Golze, J. Wilhelm, S. Chulkov, M. H. Bani-Hashemian, V. Weber, U. Borštnik, M. Taillefumier, A. S. Jakobovits, A. Lazzaro, H. Pabst, T. Müller, R. Schade, M. Guidon, S. Andermatt, N. Holmberg, G. K. Schenter, A. Hehn, A. Bussy, F. Belleflamme, G. Tabacchi, A. Glöß, M. Lass, I. Bethune, C. J. Mundy, C. Plessl, M. Watkins, J. VandeVondele, M. Krack, and J. Hutter, The Journal of Chemical Physics **152**, 194103 (2020).
[7] J. Behler and M. Parrinello, Physical Review Letters **98**, 146401 (2007).
[8] V. Kapil, M. Rossi, O. Marsalek, R. Petraglia, Y. Litman, T. Spura, B. Cheng, A. Cuzzocrea, R. H. Meißner, D. M. Wilkins, *et al.*, Computer Physics Communications **236**, 214 (2019).
[9] S. Plimpton, Journal of computational physics **117**, 1 (1995).
[10] G. Bussi, D. Donadio, and M. Parrinello, The Journal of Chemical Physics **126**, 014101 (2007).
[11] I. R. Craig and D. E. Manolopoulos, The Journal of Chemical Physics **121**, 3368 (2004).
[12] O. Marsalek and T. E. Markland, The journal of physical chemistry letters **8**, 1545 (2017).
[13] R. L. Benson, G. Trenins, and S. C. Althorpe, Faraday Discussions **221**, 350 (2019).
[14] M. Guidon, J. Hutter, and J. VandeVondele, Journal of Chemical Theory and Computation **6**, 2348 (2010).
[15] M. Pizzochero, F. Ambrosio, and A. Pasquarello, Chemical Science (2019).
[16] F. Ambrosio, G. Miceli, and A. Pasquarello, The Journal of Physical Chemistry Letters **8**, 2055 (2017).
[17] M. Guidon, J. Hutter, and J. VandeVondele, Journal of Chemical Theory and Computation **5**, 3010 (2009).
[18] S. Goedecker, M. Teter, and J. Hutter, Physical Review B **54**, 1703 (1996).
[19] C. Hartwigsen, S. Gœdecker, and J. Hutter, Physical Review B **58**, 3641 (1998).
[20] M. Del Ben, M. Schönherr, J. Hutter, and J. VandeVondele, The Journal of Physical Chemistry Letters **4**, 3753 (2013).
[21] G. Imbalzano, A. Anelli, D. Giofré, S. Klees, J. Behler, and M. Ceriotti, The Journal of Chemical Physics **148**, 241730 (2018).
[22] J. VandeVondele and J. Hutter, The Journal of Chemical Physics **127**, 114105 (2007).
[23] K. H. Schmidt, P. Han, and D. M. Bartels, The Journal of Physical Chemistry **96**, 199 (1992).
[24] K. H. Schmidt, P. Han, and D. M. Bartels, The Journal of Physical Chemistry **99**, 10530 (1995).
[25] K. H. Schmidt and W. L. Buck, Science **151**, 70 (1966).
[26] P. Gasparotto and M. Ceriotti, The Journal of Chemical Physics **141**, 174110 (2014).
[27] P. Gasparotto, R. H. Meißner, and M. Ceriotti, Journal of Chemical Theory and Computation **14**, 486 (2018).
[28] M. Ceriotti, B. A. Helfrecht, F. Giberti, and P. Gasparotto, Frontiers in molecular biosciences **6**, 24 (2019).
[29] P. Gasparotto, A. A. Hassanali, and M. Ceriotti, Journal of Chemical Theory and Computation **12**, 1953 (2016).
[30] M. Ceriotti, G. A. Tribello, and M. Parrinello, J. Chem. Theory Comput. **9**, 1521 (2013).
[31] G. A. Tribello and P. Gasparotto, in *Biomolecular Simulations* (Springer, 2019) pp. 453–502.
[32] A. Lange and J. M. Herbert, Journal of Chemical Theory and Computation **3**, 1680 (2007).
[33] E. J. Hart and J. W. Boag, Journal of the American Chemical Society **84**, 4090 (1962).
[34] J. M. Herbert, Physical Chemistry Chemical Physics **21**, 20538 (2019).
[35] M. C. Cavanagh, I. B. Martini, and B. J. Schwartz, Chemical Physics Letters **396**, 359 (2004).
