# Supplementary material for: Simulating the ghost: quantum dynamics of the solvated electron
Source: Nat Commun. 2021 Feb 3;12:766. doi: 10.1038/s41467-021-20914-0 (PMC7859219; doi:10.1038/s41467-021-20914-0)
Supplement: Supplementary file 3 — Description of Additional Supplementary Files [file 41467_2021_20914_MOESM3_ESM.pdf]

Description of Additional Supplementary Files

File Name: Supplementary Movie 1
Description: Localization of the solvated electron from quantum dynamics

File Name: Supplementary Movie 2
Description: transient diffusion of the solvated electron from quantum dynamics
